# An enrichment method based on synergistic and reversible covalent interactions for large-scale analysis of glycoproteins

**DOI:** 10.1038/s41467-018-04081-3

**Published:** 2018-04-27

**Authors:** Haopeng Xiao, Weixuan Chen, Johanna M. Smeekens, Ronghu Wu

**Affiliations:** 1 School of Chemistry and Biochemistry, Georgia Institute of Technology, Atlanta, GA 30332 USA; 2 The Petit Institute for Bioengineering and Bioscience, Georgia Institute of Technology, Atlanta, GA 30332 USA

## Abstract

Protein glycosylation is ubiquitous in biological systems and essential for cell survival. However, the heterogeneity of glycans and the low abundance of many glycoproteins complicate their global analysis. Chemical methods based on reversible covalent interactions between boronic acid and glycans have great potential to enrich glycopeptides, but the binding affinity is typically not strong enough to capture low-abundance species. Here, we develop a strategy using dendrimer-conjugated benzoboroxole to enhance the glycopeptide enrichment. We test the performance of several boronic acid derivatives, showing that benzoboroxole markedly increases glycopeptide coverage from human cell lysates. The enrichment is further improved by conjugating benzoboroxole to a dendrimer, which enables synergistic benzoboroxole–glycan interactions. This robust and simple method is highly effective for sensitive glycoproteomics analysis, especially capturing low-abundance glycopeptides. Importantly, the enriched glycopeptides remain intact, making the current method compatible with mass-spectrometry-based approaches to identify glycosylation sites and glycan structures.

## Abstract (web summary)

Understanding the functions of protein glycosylation critically depends on methods to efficiently enrich glycoproteins from complex samples. Here, the authors develop a strategy using dendrimer-conjugated benzoboroxole to enhance glycopeptide enrichment, providing the basis for more comprehensive glycoprotein analyses.

## Introduction

Glycosylation is one of the most common and essential protein modifications in cells. It often determines protein folding, trafficking, and stability, and regulates many cellular events, especially cell–cell communication, cell–matrix interactions, and cellular response to environmental cues^1–4^. Glycoproteins contain a wealth of information related to cellular developmental and diseased statuses^5,6^, and aberrant protein glycosylation is directly related to human disease, including cancer and infectious diseases^7–10^. Global analysis of protein glycosylation is critical in understanding glycoprotein functions and identifying glycoproteins as biomarkers and drug targets^10–12^. However, due to the low abundance of many glycoproteins and heterogeneity of glycans, it is extraordinarily challenging to comprehensively analyze glycoproteins in complex biological samples.

Currently, mass spectrometry (MS)-based proteomics provides a unique opportunity to globally analyze protein modifications^13–22^, including glycosylation^23–31^. However, effective enrichment prior to MS analysis is imperative for each type of protein modification. For example, with the maturity of phosphoprotein enrichment methods, the global analysis of protein phosphorylation has advanced tremendously, from the identification of several hundred phosphorylation sites a decade ago to over 10,000 sites in recent studies^32–34^.

In order to comprehensively analyze protein glycosylation in complex biological samples, several glycoprotein/glycopeptide enrichment methods have been reported, including lectin-based^35,36^ and hydrazide chemistry-based methods^37,38^, and hydrophilic interaction liquid chromatography (HILIC)^39,40^. Currently, lectin-based methods are most commonly used to enrich glycopeptides prior to MS analysis. Due to the inherent specificity of lectins, each type of lectin can only recognize a specific glycan structure, and thus, no single lectin or a combination of several lectins can universally enrich all glycosylated peptides or proteins. HILIC has also been extensively used to enrich glycopeptides based on their increased hydrophilicity by glycans. However, this method lacks specificity because it cannot distinguish glycopeptides from many hydrophilic non-glycopeptides. Recently, two elegant methods, i.e., isotope-targeted glycoproteomics (IsoTaG)^41^ and solid-phase extraction of *N*-linked glycans and glycosite-containing peptides (NGAG)^42^, have been reported. By using IsoTaG, 32 *N*-glycopeptides and over 500 intact and fully elaborated *O*-glycopeptides from 250 proteins across three human cell lines were identified^41^. NGAG was beautifully designed for *N*-glycopeptide enrichment, and 2044 unique *N*-glycopeptides were identified in mammalian cells^42^. According to prediction and computational results, protein glycosylation is the most common modification^43,44^. Despite the considerable progress that has been made in the past decade^35,37,41,42,45–50^, there is still a substantial gap between the number of glycoproteins reported in the literature and those existing in complex biological samples. Effective enrichment of glycopeptides/glycoproteins will profoundly advance the global analysis of protein glycosylation through MS-based proteomics.

Previously, boronic acid (BA) was demonstrated to have great potential in universally enriching glycopeptides for the global analysis of protein glycosylation because of its reversible covalent interactions with glycans^51,52^. However, the method suffers from relatively weak interactions; therefore, low-abundance glycoproteins are not effectively enriched. In this work, we develop an effective method to enrich glycopeptides, especially those of low abundance, by greatly enhancing the interactions between BA and glycopeptides. First, different boronic acid derivatives are tested, and benzoboroxole is found to be highly effective to enrich glycopeptides due to dramatically strengthened interactions. Second, based on the common features of a glycan containing multiple monosaccharides and one sugar bearing several hydroxyl groups, benzoboroxole-conjugated dendrimer beads are used to synergistically interact with glycopeptides. The experimental results demonstrate that conjugating benzoboroxole to a dendrimer significantly increases the enrichment efficiency, even for glycopeptides only containing β-linked *N*-acetylglucosamine (*O*-GlcNAc).

The method is applied for the global analysis of glycoproteins in yeast (*Saccharomyces cerevisiae*), mouse brain tissue, and human cells (MCF7, HEK 293T, and Jurkat). Over 1000 *N*-glycosylation sites in yeast, 4195 sites on 1608 *N*-glycoproteins in mouse brain tissues, and 4691 sites on 1906 *N*-glycoproteins in human cells are identified, including many proteins with low abundance. The reversible nature of the interactions allows us to analyze intact *O*-glycopeptides with glycan structure information. We identify 234 *O*-mannosylated proteins in yeast and many glycoproteins with *O*-GlcNAc in human cells. These results demonstrate that the current method is universal and highly effective in enriching glycopeptides, especially from low-abundance glycoproteins that are normally of greater biological importance. The current results also provide valuable information regarding glycoproteins in yeast and human cells to biological and biomedical research communities. Without sample restrictions, this method can be applied to many other samples for glycoprotein analysis.

## Results

### Enhancing glycopeptide enrichment with BA derivatives

Boronic acid can form reversible covalent bonds with sugars and has been extensively used for sugar detection^53–55^. Therefore, BA-based methods have great potential in universally enriching glycopeptides and glycoproteins, and the reversible nature of the interaction leaves enriched glycopeptides intact after the release. However, the interaction between BA and sugar is relatively weak, preventing the enrichment of low-abundance glycoproteins. To effectively enrich low-abundance glycoproteins, which often contain important information, it is critical to strengthen the interaction.

**Fig. 1:**
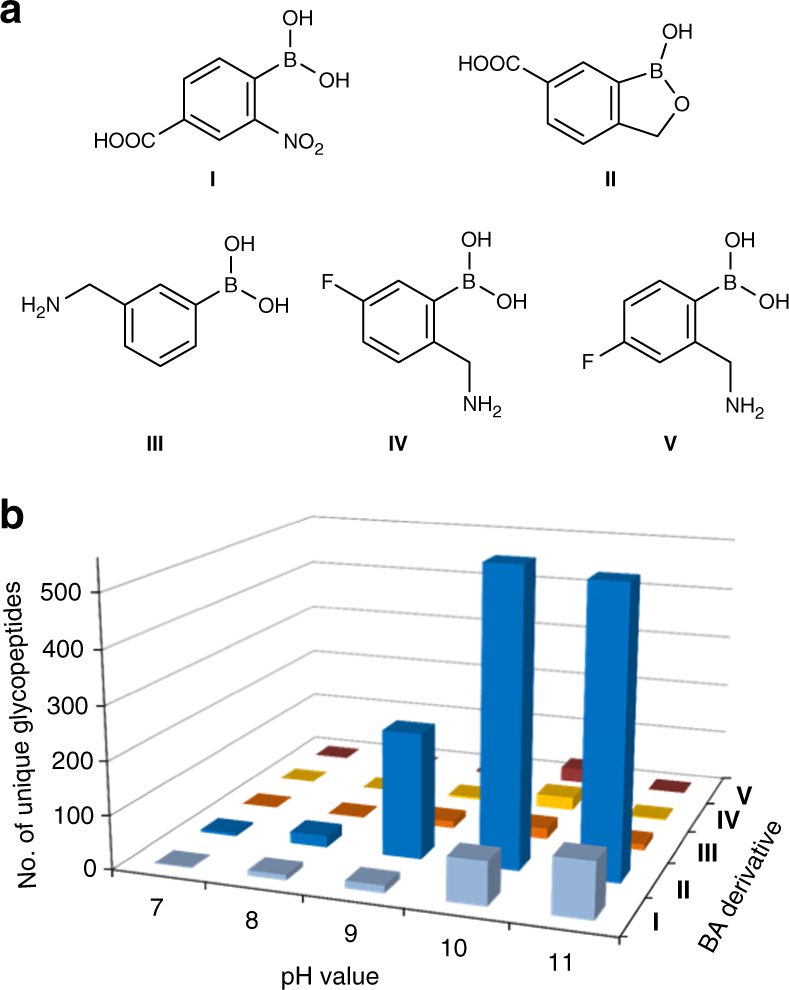
Structures of boronic acid derivatives and experimental results using different derivatives. **a** Structures of boronic acid derivatives tested in this work. **b** The number of unique *N*-glycopeptides identified with each BA derivative at varying pH values from the parallel experiments

Previously, we employed phenylboronic acid-conjugated beads to enrich glycopeptides from yeast whole-cell lysates for global analysis^52^. In yeast, high-mannose glycans dominate, while in mammalian cells, glycans are more structurally diverse. To effectively cover low-abundance glycoproteins and those containing highly diverse glycans, we have redesigned a BA-based method. First, we attempted to enhance the interactions by examining several boronic acid derivatives. The structures of BA derivatives tested here are displayed in Fig. 1a. In parallel experiments starting with the same amount of purified peptides from human cells (HEK 293T), enrichment with derivatives **I**, **IV**, and **V** resulted in slightly more unique *N*-glycopeptides compared to phenylboronic acid (**III**) (Fig. 1b), which was used previously^52^ (more details discussed in Supplementary Note 1).

Among these five boronic acids tested, derivative **II** (benzoboroxole) allowed the identification of the greatest number of glycopeptides. The interactions between benzoboroxole and sugars were reported to be stronger than those between phenylboronic acid and sugars^54,56,57^. For example, the binding constant (*K*_a_) for the reaction between benzoboroxole and fructose is 606 M^−1^ at neutral pH, which is many times higher than that between phenylboronic acid and fructose (79 M^−1^) under identical conditions^56^. The current experimental results are very consistent with previous findings, and stronger interactions between BA and glycans can more effectively enrich glycopeptides. The method based on benzoboroxole was systematically optimized for site-specific and global analysis of glycoproteins in combination with MS, and the results were dramatically improved compared to any other boronic acids tested here.

### Synergistic interactions to increase glycopeptide coverage

Strengthening the interactions between benzoboroxole and glycopeptides will further increase the coverage of low-abundance glycopeptides. One glycan typically contains multiple monosaccharides, which allows one glycopeptide to interact with multiple benzoboroxole molecules. The synergistic effect for the interactions between multiple BA derivative molecules and glycans is expected to further facilitate the enrichment of glycopeptides, especially those with low abundance. Here, we synthesized a dendrimer as the platform for synergistic interactions because the number of benzoboroxole molecules bound to a dendrimer can be easily adjusted. More importantly, the dendrimer branches also provide structural flexibility to enhance the synergistic interactions.

**Fig. 2:**
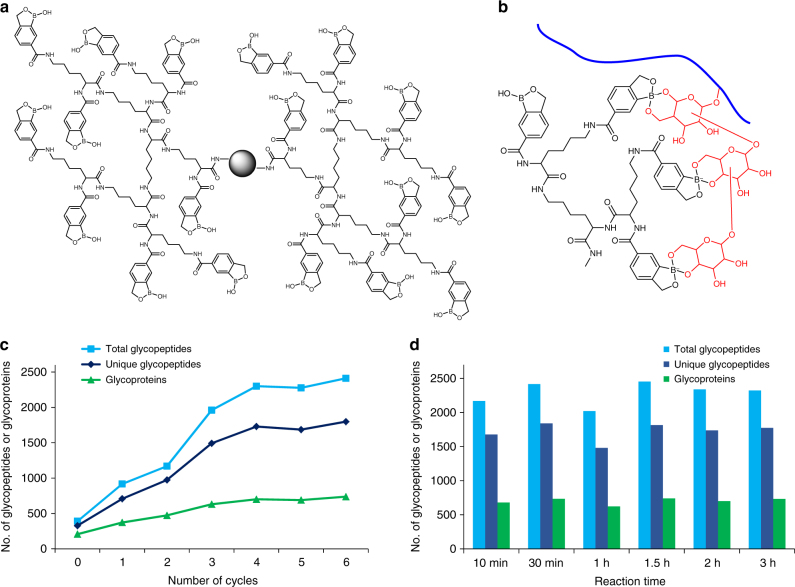
Principal and experimental results of the synergistic interactions between the DBA beads and several sugars from one glycopeptide. **a** The structure of a dendrimer conjugated with BA derivative **II** (benzoboroxole). **b** An example of the synergistic interactions between multiple benzoboroxole molecules in a dendrimer and several sugars within one glycan of a glycopeptide. **c** The effect of synthesis cycles and the corresponding dendrimer size on the enrichment of glycopeptides: total glycopeptides (blue), unique glycopeptides (dark blue), and glycoproteins (green) identified in parallel experiments. **d** The effect of reaction time on the *N*-glycopeptide identification

The dendrimer was first synthesized and bound to magnetic beads, and next, the BA derivative, benzoboroxole, was conjugated to the dendrimer (Supplementary Figs. 1 and 2). Many benzoboroxole molecules were bound to one dendrimer, as shown in Fig. 2a, and the number of benzoboroxole molecules on one dendrimer bead is proportional to the dendrimer size. In this case, several sugars from one glycan may interact with multiple benzoboroxole molecules simultaneously (Fig. 2b).

Dendrimer size is expected to have a large impact on the synergistic interactions, and the effect of dendrimer size was systematically evaluated. For the parallel experiments, the number of benzoboroxole molecules on the beads attempted to remain the same, and the amount of starting materials (peptides from HEK 293T cells) was also the same. In Fig. 2c, when the cycle number is zero, the magnetic beads are directly conjugated with benzoboroxole without a dendrimer. The dendrimer size increases with the number of rounds of synthesis, as well as the number of benzoboroxole molecules after conjugation. With dendrimer beads synthesized through one to four rounds of the reaction, the number of total *N*-glycopeptides, unique *N*-glycopeptides, and *N*-glycoproteins increased linearly (Fig. 2c). After four rounds of synthesis, the numbers are very comparable, and the specificity results have a similar trend (Supplementary Fig. 3). Once the number of benzoboroxole molecules on a single bead reaches the threshold, larger dendrimers with more benzoboroxole molecules do not affect the synergistic interactions, which occurs after four rounds of synthesis.

Since the enrichment reaction is quick and the conditions are mild, prolonging the reaction time does not have negative effects on glycopeptide identification. As shown in Fig. 2d, a similar number of unique *N*-glycopeptides and glycoproteins were identified when the incubation time varied from 10 min to 3 h. A more detailed description and results from further optimization are provided in Supplementary Figs. 4–7 and Supplementary Note 2. We also assessed the residual *N*-glycans after peptide-*N*-glycosidase F (PNGase F) treatment, and duplicate experiments were performed to examine the percentage of residual *N*-glycans. The results demonstrated that the *N*-glycan removal efficiency with PNGase F within 3 h was very high (Supplementary Fig. 8 and Supplementary Note 3).

### Comparing with existing lectin- and HILIC-based methods

**Fig. 3:**
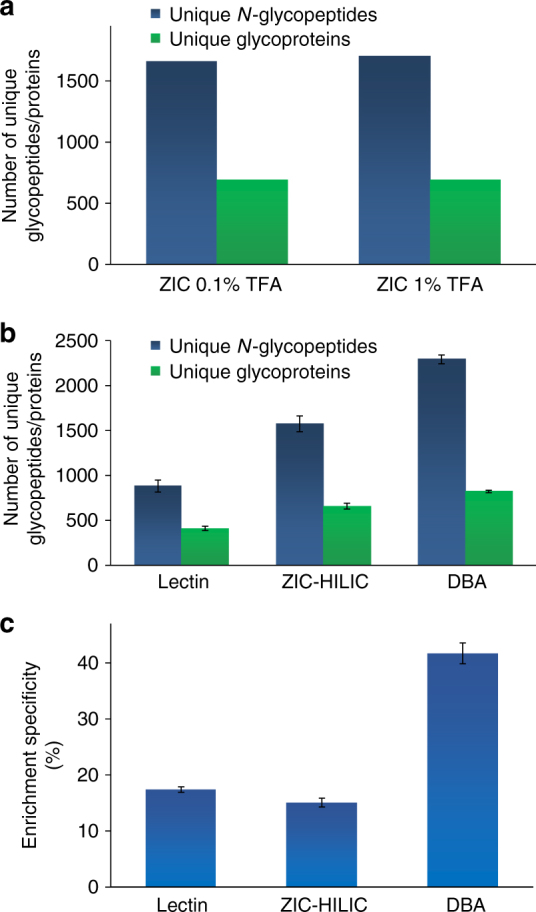
Comparison of three enrichment methods. **a** Optimization of the concentrations of TFA as the ion-pairing reagent for ZIC-HILIC enrichment. **b** The numbers of unique glycopeptides (dark blue) and glycoproteins (green) identified using each of the three methods (lectin, ZIC-HILIC, and DBA) from parallel experiments. **c** Comparison of the enrichment specificity for three enrichment methods. The error bar represents the standard error of the mean calculated from triplicate experiments

To test the effectiveness of the Dendrimer-conjugated Boronic Acid derivative (DBA) enrichment, triplicate parallel experiments were performed to compare the current method with the commonly used lectin (combining wheat germ agglutinin (WGA) and concanavalin A (ConA)) and zwitterionic HILIC (ZIC-HILIC) enrichment methods. Each experiment started from the same amount of peptides from an MCF7 cell whole lysate (Fig. 3). For these parallel experiments, except the enrichment method, every other step was kept the same. Prior to this comparison, we compared 0.1% and 1% trifluoroacetic acid (TFA) as the ion-pairing reagent for the ZIC-HILIC experiment and found that 1% TFA had slightly better performance (Fig. 3a). Therefore, we used 1% TFA in the comparison experiment. From the parallel experiments, the greatest number of unique *N*-glycopeptides were identified using the current DBA method, and more unique *N*-glycopeptides were identified with ZIC-HILIC than the lectin-based method (Fig. 3b).

Regarding the specificity, we compared the numbers of glycopeptides and non-glycopeptides identified in each of the parallel experiments, and the results showed that the DBA method had the highest specificity (Fig. 3c). We reasoned that although ZIC-HILIC allows for enrichment of a broader spectrum of glycopeptides than lectin, the principle of the ZIC-HILIC method is based on the hydrophilic property difference between glycopeptides and non-glycopeptides. Therefore, some hydrophilic but non-glycosylated peptides can also be enriched, lowering the enrichment specificity. Based on the number of unique glycopeptides identified, DBA outperformed the other two methods, while ZIC-HILIC had better performance than lectin. Furthermore, the current method also has the highest enrichment specificity.

### Global characterization of protein *N*-glycosylation in yeast

**Fig. 4:**
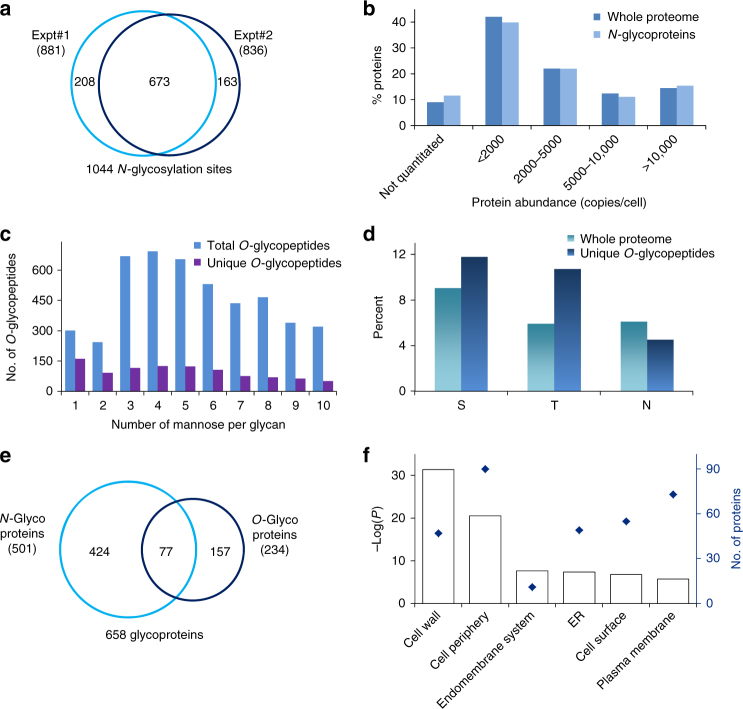
Comprehensive analysis of protein *N*- and *O*-glycosylation in yeast. **a** Protein *N*-glycosylation sites identified in biological duplicate experiments. **b** Abundance distributions of the whole proteome (dark blue) and *N*-glycoproteins identified here (light blue). **c** Distribution of the number of mannose residues per glycan on all identified *O*-glycopeptides. **d** Percentages of S, T, and N in *O*-glycopeptides compared to the whole proteome. **e** Comparison of *O*- and *N*-glycoproteins identified in yeast cells. **f** Clustering of *O*-glycoproteins based on the cellular compartment. *P* values are calculated by a modified Fisher’s exact test^61^

Using the current method, we performed biological duplicate experiments for the global analysis of protein *N*- and *O*-glycosylation in yeast. For *N*-glycoprotein analysis, we identified 881 sites on 400 proteins in one experiment (Supplementary Data 1a) and 836 sites on 404 proteins in the other (Supplementary Data 1b). Overall, 1044 *N*-glycosylation sites (Fig. 4a and Supplementary Data 1c) on 501 proteins (Supplementary Fig. 9) were identified. To ensure that the sites were confidently identified, very stringent criteria were applied during analysis. First, the false-positive rate at the *N*-glycopeptide level was well-controlled under 1.0%, based on the target–decoy method^58^. Additionally, all *N*-glycosylation sites were required to contain the motif NX[S/T/C], where X is any amino acid except proline. The *N*-glycosylation site was also required to contain heavy oxygen (^18^O) as a tag^45^. To minimize possible spontaneous deamidation during PNGase F treatment in heavy-oxygen water, the reaction was run for only 3 h. Our previous results demonstrated that within 3 h under mild conditions, spontaneous asparagine deamidation is negligible^59^.

In order to demonstrate that low-abundance glycoproteins can be identified with the current method, we compared the abundance distributions of identified *N*-glycoproteins and all proteins in the whole-yeast proteome, and they were very similar (Fig. 4b). We reanalyzed our previous data set using phenylboronic acid magnetic beads in yeast^52^ with the same criteria as above, and 716 *N*-glycosylation sites on 297 proteins were identified. The abundance distributions for both data sets are shown in Supplementary Fig. 10. More *N*-glycoproteins were identified in each bin with the current method, especially for low-abundance *N*-glycoproteins (abundance from the literature^60^). For example, for proteins with abundances less than 2000 copies per cell, about twice as many *N*-glycoproteins were identified in this work (158 vs. 84), which clearly demonstrated that the current method is more effective in enriching low-abundance glycopeptides due to strengthened interactions from the BA derivative and synergistic interactions of DBA.

### Analyzing protein *O*-mannosylation in yeast

The reversible covalent interactions can leave enriched glycopeptides with intact glycans for site identification and glycan structure elucidation. In baker’s yeast, *O*-glycans consist of only mannose, but the number of mannose per glycan varies. The current enrichment method also enables us to globally analyze *O*-glycoproteins. In order to increase the identification confidence of intact *O*-glycopeptides, high-energy collisional dissociation (HCD) was employed for glycopeptide fragmentation, and the tandem mass spectra were recorded in the Orbitrap cell. Several important machine parameters, such as automatic gain control (AGC) target for MS and MS^2^, normalized collision energy, and maximum ion accumulation time for MS^2^, were optimized (Supplementary Fig. 11). We used Byonic^TM^ to search the raw files for the identification of protein *O*-mannosylation.

Several examples of the *O*-mannosylated peptides with different glycans identified here are displayed in Supplementary Fig. 12. Here, we identified 987 unique *O*-glycopeptides from 206 proteins (Supplementary Data 2a) in the first experiment and 971 unique *O*-glycopeptides from 196 proteins (Supplementary Data 2b) in the second experiment. In total, 234 *O*-glycoproteins were identified, and 168 proteins were identified in both experiments. The overlap was very high (81.6 and 85.7%), which further demonstrated that the identification of glycopeptides and glycoproteins was highly confident. The current results are a proof-of-concept to show that the glycopeptide enrichment based on the reversible covalent interactions can keep enriched glycopeptides intact for site identification and glycan structure elucidation.

The distribution of the number of mannose per glycan is shown in Fig. 4c. The number of unique glycopeptides with one mannose is the highest, and the second are those with four mannoses. For glycopeptides with glycans containing more than four mannoses, the number decreases with the increasing number of mannoses. The site localization confidence is lower than that of *N*-glycosylation due to the neutral loss of *O*-glycans and the presence of many serine and threonine residues on *O*-glycopeptides (Fig. 4d). Compared to the whole-yeast proteome, both S and T were more frequent in the identified unique *O*-glycopeptides, and the occurrence of T was almost two times as many (9.0 vs. 11.8% for S and 5.9 vs. 10.7% for T). Conversely, the frequency of N (*N*-glycosylation sites) in the identified *O*-glycopeptides was lower than the whole yeast proteome (6.1 vs. 4.5%).

In total, 234 *O*-glycoproteins (Supplementary Data 2c) were identified, and about one-third were also *N*-glycosylated (Fig. 4e). *O*-glycoproteins located on the cell wall (*P* = 4.25E−32, modified Fisher’s exact test^61^, which is also used for all other *P *value calculations except those stated otherwise) are the most highly enriched when clustered using the Database for Annotation, Visualization, and Integrated Discovery (DAVID)^61^ (Fig. 4f). Seventy-three *O*-glycoproteins belong to the endomembrane system, and 55 are located in the ER. Clustering of *O*-glycoproteins based on molecular function indicates that proteins related to hydrolase activity (acting on glycosyl bonds) and transferase activity (transferring glycosyl groups) are most highly enriched (Supplementary Fig. 13). Based on reversible covalent interactions between DBA and glycans, protein *O*-glycosylation can be confidently identified, including valuable glycan structural information.

### Global analysis of protein *N*-glycosylation in human cells

**Fig. 5:**
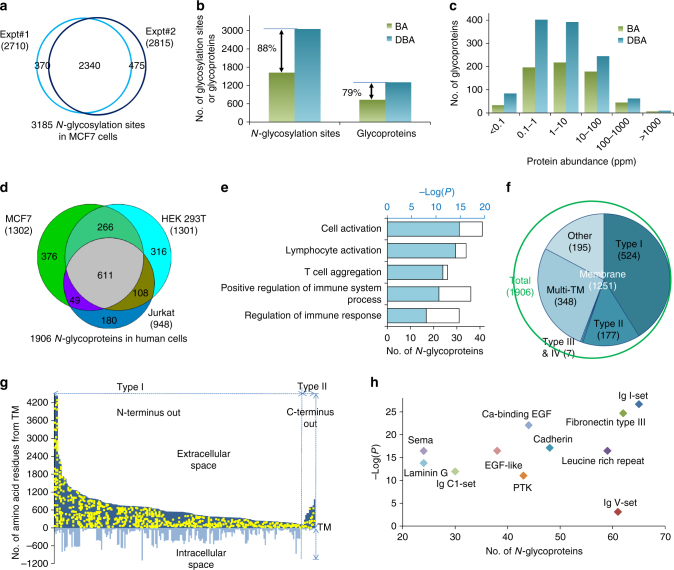
Comprehensive analysis of protein *N*-glycosylation in human cells. **a** Comparison of unique protein *N*-glycosylation sites identified in MCF7 cells in duplicate experiments. **b** Comparison of unique glycosylation sites and glycoproteins identified with the boronic acid derivative magnetic beads (designated as BA, shown in green) and with the dendrimer beads conjugated with the boronic acid derivative (DBA) (blue). **c** Abundance distributions of *N*-glycoproteins identified with the BA (green) or DBA (blue) beads. **d** Overlap of *N*-glycoproteins in three different types of cells (MCF7, HEK 293T, and Jurkat). **e** Protein-clustering results for 180 *N*-glycoproteins identified exclusively in Jurkat cells. **f** Distribution of membrane proteins (type I, II, III, and IV, and multi-pass transmembrane (TM)) among all identified *N*-glycoproteins. **g** *N*-glycosylation site locations on 301 receptors with *X*-axis as the TM domain. Each glycoprotein sequence was aligned against the transmembrane domain, and the glycosylation sites are indicated as yellow dots. All sites are located in the extracellular space. **h** Domain analysis of *N*-glycoproteins showing the number of *N*-glycoproteins containing the most highly enriched domains and their corresponding *P* values

Due to the diversity of glycan structures, it is more challenging to globally analyze glycoproteins in human cells. The DBA method was applied to comprehensively analyze protein *N*-glycosylation in different types of human cells. Biological duplicate experiments were performed for MCF7 cells, and the number of glycosylation sites and glycoproteins identified in each experiment is shown in Fig. 5a and Supplementary Fig. 14. With the well-controlled false-discovery rate (FDR) of <1.0% at the glycopeptide level and stringent criteria described above, we identified 2710 *N*-glycosylation sites on 1127 proteins (different isoforms are not counted) in one experiment (Supplementary Data 3a), and 2815 sites on 1156 proteins in the other (Supplementary Data 3b). Overall, 2340 common sites were identified in both experiments, which represent 86.3% and 83.1% of the total sites identified from each experiment, respectively. As expected, the overlap at the glycoprotein level was even higher: 981 common glycoproteins were identified. A total of 3185 glycosylation sites were identified on 1302 proteins in MCF7 cells (Supplementary Data 3c).

The method was also employed to globally analyze protein *N*-glycosylation in HEK 293T and Jurkat cells; 3052 sites were identified on 1301 proteins in HEK 293T cells (Supplementary Data 4), and 2120 sites on 948 proteins were found in Jurkat cells (Supplementary Data 5). The comparison of identified sites is shown in Supplementary Fig. 15.

We further tested the effect of the dendrimer on glycopeptide enrichment by comparing DBA vs. benzoboroxole-conjugated magnetic beads without the dendrimer (designated as BA beads). With the DBA beads, we were able to identify 88% more *N*-glycosylation sites and 79% more glycoproteins compared to the BA beads (Fig. 5b). The abundance distributions of all glycoproteins identified using either the DBA or BA beads are displayed in Fig. 5c (abundances from an online database (PaxDb)^62^). Besides, the number of glycoproteins identified using the DBA beads was higher than that with the BA beads in each abundance category, and the DBA method was especially superior for glycoproteins with very low abundance (less than 10 ppm). For low-abundance proteins, over twice as many *N*-glycoproteins were identified with the DBA beads (84 vs. 34 glycoproteins for <0.1 ppm, and 402 vs. 196 for 0.1–1.0 ppm). These results explicitly demonstrate that the synergistic interactions between multiple BA derivative molecules and glycans can greatly increase the coverage of low-abundance glycopeptides.

Combining the results from the three human cell lines, we identified a total of 4691 *N*-glycosylation sites (Supplementary Data 6) on 1906 proteins (Fig. 5d, and Supplementary Data 7). More than 10% of proteins (238) are highly glycosylated and contain at least five sites (Supplementary Fig. 16). In consideration of different cell types, there is a decent overlap among identified *N*-glycoproteins in human cell experiments (Fig. 5d). One example highlighting the differences between cell types are the *N*-glycoproteins (180) identified only in Jurkat cells, many of which are related to immune cell-specific activities, such as cell activation and cell's immune response (Fig. 5e).

By clustering 1906 *N*-glycoproteins according to their molecular function, proteins related to glycosyltransferase activity are the most highly enriched with a *P* value of 8.5E−35 (Supplementary Fig. 17a), and 108 *N*-glycoproteins belong to this category. In yeast, this group of proteins is most highly enriched as well. The following groups of *N*-glycoproteins are also highly enriched in human cells: receptor binding, signaling receptor activity, growth factor-binding proteins, glycosaminoglycan binding, cell adhesion molecule binding, and active transmembrane transporter activity.

Many glycoproteins are known to be membrane proteins. Here, 1251 out of 1906 *N*-glycoproteins are membrane proteins, which are highly enriched with an extremely low *P* value of 1.6E−192. Glycoproteins in the cell periphery, vesicle, ER, Golgi, and extracellular space are all enriched with very low *P* values (Supplementary Fig. 17b). Based on the information available on UniProt (uniprot.org), 524 of identified membrane proteins are type-I membrane proteins, 177 are type II, and 348 proteins contain multiple transmembrane domains (Fig. 5f). A total of 301 receptors were identified among these *N*-glycoproteins (Supplementary Fig. 18); glycosylation site locations for receptors identified as type-I and type-II membrane proteins are shown in Fig. 5g. All sites (1079 sites, Supplementary Data 8) were located in the extracellular space, which corresponds very well with the belief that glycans are located on the extracellular side of surface membrane proteins.

Domain analysis shows that many *N*-glycoproteins contain different types of Ig domains (such as I-set, V-set, and C1-set). Besides Ig domains, other domains related to cell–cell adhesion are also highly enriched, including fibronectin type III, cadherin, and laminin G (Fig. 5h). Domains corresponding with receptor activities, such as PTK (protein tyrosine kinase) and EGF (epidermal growth factor)-like domains, are also highly enriched.

### Analysis of protein *N*-glycosylation in mouse brain tissues

Without sample restriction, the current method can be applied for glycoprotein analysis in any other samples, including animal tissues and clinical samples. Here, we further applied this method to analyze protein *N*-glycosylation in mouse brain tissues, and biological duplicate experiments were performed. After protein extraction and digestion, glycopeptides were enriched using the DBA beads, and enriched glycopeptides were fractionated, followed by analysis with an online LC–MS system.

In the first experiment, we identified 3583 sites on 1434 glycoproteins (Supplementary Data 9a), and very similar results were obtained in the second experiment (3685 sites on 1443 proteins) (Supplementary Data 9b). In total, 4195 sites were identified on 1608 proteins (Supplementary Data 9c), and 3073 common sites and 1269 glycoproteins were found in both experiments, as shown in Supplementary Fig. 19. Considering the large-scale analysis and the experiments being biologically duplicate, the overlap is very high at both the site (85.8 and 83.4% compared to both the experimental results, respectively) and protein (88.5 and 87.9%) levels, which is consistent with the above results from the duplicate experiments in human cells. The highly reproducible results further demonstrate that the current method is effective.

Glycoproteins identified in the mouse brain tissues were clustered using DAVID based on biological processes. About one-quarter of identified glycoproteins (396) are related to cell surface receptor signaling pathway, which is the most highly enriched with a *P* value of 1.1E−61. Proteins related to brain-specific functions such as nervous-system development (*P* = 4.1E−61), axon development (*P* = 1.9E−54), and synapse assembly (*P* = 2.6E−30) were also highly enriched, as shown in Supplementary Fig. 20. *P* values are calculated by a modified Fisher’s exact test^61^.

### Synergistic interactions to identify protein *O*-GlcNAcylation

Protein *O*-GlcNAcylation was discovered more than three decades ago, and it has been reported to be involved in many cellular events, from regulating cell signaling to gene expression^63–65^. Using BA-based methods, it is challenging to enrich *O*-GlcNAcylated proteins because only one sugar (GlcNAc) is bound to S or T, and this sugar does not contain a *cis*-1,2-diol. Although boronic acid can interact with sugars without *cis*-1,2-diols, such as glucose and GlcNAc, the interaction is weak^53^, and enrichment is therefore less effective.

**Fig. 6:**
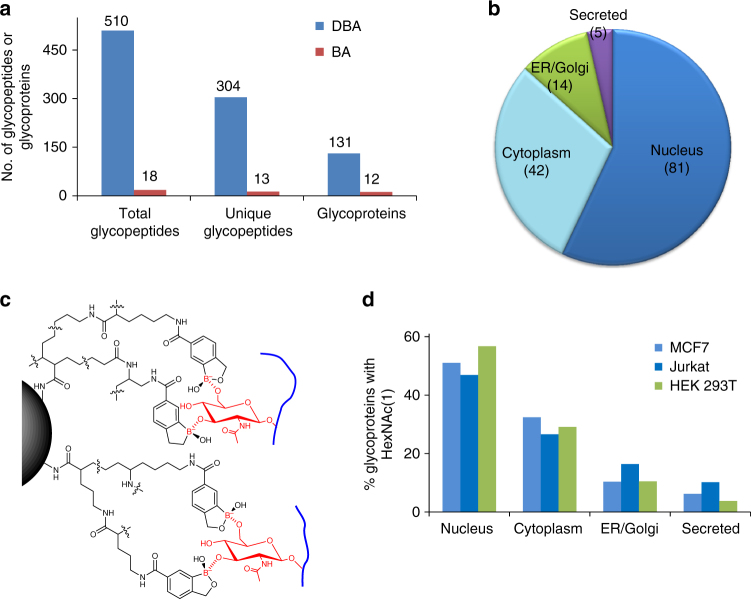
The synergistic interactions dramatically enhanced the enrichment of *O*-GlcNAcylated peptides in human cells. **a** Comparison of glycoproteins with one HexNAc identified with BA and DBA, which clearly shows that the results from DBA are substantially better. **b** Distribution of *O*-glycoproteins modified with HexNAc(1) identified in HEK 293T cells based on the cellular compartment. **c** Proposed mechanism of the interactions between DBA and GlcNAc benefiting from synergistic interactions. **d** Cellular compartment distributions of glycoproteins containing one HexNAc identified in the three types of human cells (light blue—MCF7, dark blue—Jurkat, and green—HEK 293T)

In this work, we identified 510 total glycopeptides with *N*-acetylhexosamine (HexNAc) (1) and 304 unique glycopeptides located on 131 proteins in HEK 293T cells with the DBA enrichment (Fig. 6a). In striking contrast, with the BA derivative magnetic beads, only 18 total glycopeptides with HexNAc and 13 unique glycopeptides were found on 12 proteins. Among 131 glycoproteins, 81 were located in the nucleus (Fig. 6b), and typically, these proteins are *O*-GlcNAcylated because only glycoproteins with *O*-GlcNAc have been reported in the nucleus. Similarly, 131 *O*-glycoproteins with HexNAc(1) were identified in MCF7 cells, and 119 *O*-glycoproteins were found in Jurkat cells (Supplementary Data 10).

The effective enrichment of *O*-GlcNAcylated peptides may be attributed to the synergistic interactions with DBA beads. As discussed above, multiple sugars from one glycan synergistically interact with different benzoboroxole molecules on a single dendrimer bead. Although there is no *cis*-1,2-diol in GlcNAc, multiple hydroxyl groups in each GlcNAc may form reversible covalent bonds with several benzoboroxole molecules on a dendrimer bead, as shown in Fig. 6c. The synergistic interactions can dramatically facilitate the enrichment of *O*-GlcNAcylated peptides with DBA. The results are highly reproducible in different types of human cells (HEK 293T, MCF7, and Jurkat), and glycoproteins with one HexNAc are listed in Supplementary Data 10. The greatest number of identified glycoproteins (about 50%) are located in the nucleus of each cell type (Fig. 6d), and about 30% of them are in the cytoplasm. Glycoproteins in the nucleus and the cytoplasm are normally *O*-GlcNAcylated. In addition, ~12% of them are in the ER/Golgi. Only a small portion of glycoproteins (~7%) are secreted proteins, which are likely *O*-GalNAcylated.

## Discussion

Based on universal and reversible covalent interactions between boronic acid and sugars, BA-based enrichment methods have great potential in enriching glycopeptides for global analysis of protein glycosylation. However, the relatively weak interactions prevent the enrichment of glycopeptides with low abundance. In order to effectively enrich glycopeptides in complex biological samples, it is critical to strengthen the interactions between BA and glycans.

In this work, we enhanced the interactions between BA and glycans through two ways. First, we employed the BA derivative (benzoboroxole) to form stronger interactions with glycans, which was able to dramatically increase the coverage of low-abundance glycopeptides, as shown in Fig. 1b and Supplementary Fig. 10. Second, based on the common features of a glycan containing multiple monosaccharides and one sugar bearing several hydroxyl groups, we benefited from synergistic interactions by conjugating many benzoboroxole molecules onto a dendrimer bead. The synergistic interactions between several benzoboroxole molecules on a bead and different sugars within a glycan make the enrichment much more effective, which is clearly demonstrated from the current results (Fig. 5b, c). The dendrimer provides an excellent platform to conjugate many benzoboroxole molecules onto the same bead. The dendrimer size is readily adjustable, and correspondingly, the number of benzoboroxole molecules can be controlled on each bead. Furthermore, the dendrimer provides structural flexibility to form stronger interactions with glycans.

The reversible nature of the interactions between BA and glycans allows enriched peptides to be released with intact glycans. The direct analysis of intact glycopeptides provides valuable information about protein glycosylation sites and glycan structures. We systematically analyzed *O*-mannosylated proteins and their glycan structures in yeast, and overall, 234 *O*-glycoproteins were identified. With stringent criteria for data analysis, the identifications of *O*-glycopeptides and *O*-glycoproteins are highly confident. However, compared to protein *N*-glycosylation site identification, *O*-glycosylation sites were less confidently localized because of the possible neutral loss of glycans during intact *O*-glycopeptide analysis using HCD and high percentages of S and T in glycopeptides.

Synergistic interactions can enhance not only the interactions between benzoboroxole and glycans containing multiple monosaccharides but also the interactions with *O*-GlcNAcylated peptides. It is well-known that BA can form stronger interactions with sugars containing *cis*-1,2-diols. The interaction between BA with glucose and GlcNAc without *cis*-1,2-diols is much weaker^53^. Here, due to the flexible nature of the dendrimer, one GlcNAc may form multiple covalent bonds with different benzoboroxole molecules, as shown in Fig. 6c. Compared to BA beads, DBA is much more effective in enriching *O*-GlcNAcylated peptides (Fig. 6a).

Cluster of differentiation (CD) molecules are those located on the cell surface that provide immunophenotyping targets for cell classification^66^. In our experiment, 188 CD proteins were identified as *N*-glycoproteins (Supplementary Data 11). There were more CDs identified in Jurkat cells (137) than MCF7 (115) or HEK 293T (129) cells (Supplementary Fig. 21a and b), despite the fact that the total *N*-glycoproteins identified in Jurkat cells were fewer. However, this result is consistent with the fact that more CDs are relevant to immune-related cells, including Jurkat cells. Two examples of glycoproteins identified in Jurkat cells are shown in Supplementary Fig. 22, and the majority of identified *N*-glycosylation sites are located in extracellular domains (Supplementary Note 4). CDs with site-specific information may be more meaningful for cell classification and serve as effective biomarkers for disease detection.

Benefiting from the common features of glycans, the current method can dramatically enhance the interactions between boronic acid and glycans, which is critical in analyzing glycoproteins with low abundance. Furthermore, there are several other advantages. First, this method is quick and easy to operate. As shown in Fig. 2d, the results from 10-min incubation are almost the same as those from 2- or 3-h incubation. Glycopeptides are captured under basic conditions and released in an acidic solution. Second, this method is highly reproducible and robust. Third, because the enrichment is based on the reversible interactions, the enriched glycopeptides remain intact, which allows us to analyze glycan structures and also to identify protein *O*-glycosylation, as demonstrated by the analyses of protein *O*-mannosylation in yeast and *O*-GlcNAcylation in human cells. Fourth, because there are no sample restrictions, this method can be extensively applied to analyze different types of samples, from whole-cell lysates to clinical and plant samples.

The current method is based on the universal and reversible interactions between hydroxyl groups in glycans and boronic acid. The experimental results for yeast and human cells, and mouse tissue demonstrated that this method is highly effective in enriching glycopeptides, especially for those with low abundance, and the reversible nature of the interactions keeps enriched glycopeptides intact for both site identification and glycan structure analysis. Due to the biological importance of glycoproteins, their global analysis will aid in a better understanding of glycoprotein functions and the molecular mechanisms of diseases, and the discovery of glycoproteins as drug targets and disease biomarkers.

## Methods

### Materials

Complete protease inhibitors were purchased from Roche Applied Sciences and sequencing-grade trypsin was purchased from Promega. Dulbecco’s modified Eagle’s medium (DMEM), phosphate-buffered saline (PBS), *N*-(3-dimethylaminopropyl)-*N*′-ethylcarbodiimide hydrochloride (EDC), 4-carboxy-2-nitrophenylboronic acid, (2-aminomethyl-5-fluoro) phenylboronic acid hydrochloride, 2-aminomethyl-4-fluorophenylboronic acid hydrochloride, trifluoroacetic acid, formic acid (FA), trimethylamine (TEA), piperidine, methanol, chloroform, dichloromethane (DCM), acetonitrile (ACN), and dimethylsulfoxide (DMSO) were purchased from Sigma-Aldrich. 3-aminomethylphenylboronic acid hydrochloride was purchased from Frontier Scientific Inc. 5-carboxybenzoboroxole and 1-hydroxy-7-azabenzotriazole (HOAt) were purchased from AK Scientific, Inc. (2,5-dioxopyrrolidin-1-yl) (2S)-2-(9H-fluoren-9-yl-methoxycarbonylamino)-6-[(2-methylpropan-2-yl)oxycarbonylamino] hexanoate (Fmoc-l-Lys(Boc)-OSu) and (S)-2,5-dioxopyrrolidin-1-yl 2,6-bis((tert-butoxycarbonyl) amino) hexanoate (Boc-Lys(Boc)-OSu) were purchased from Ark Pharm, Inc. and Sigma-Aldrich. MagnaBind™ amine derivatized beads, MagnaBind™ carboxyl derivatized beads, and fetal bovine serum (FBS) were bought from Thermo Fisher Scientific.

### Magnetic beads derivatization

MagnaBind^TM^ amine- (or carboxyl-) derivatized beads were washed with DMSO three times. EDC was mixed with the boronic acid derivative in DMSO and incubated end-over-end for 10 min; HOAt was subsequently added, and the reaction mixture was further incubated for 1 h. The mixture was then transferred to the beads slurry and incubated overnight in DMSO containing 3.0% triethylamine (TEA). The boronic acid functionalized beads were washed with DMSO twice and 20% ACN three times and stored in 20% ACN for further use.

For dendrimer boronic acid derivatization, the solvent containing the MagnaBind^TM^ amine derivatized beads was gradually changed from water to isopropanol to finally DCM (Supplementary Figs. 1 and 2). Then Fmoc-l-Lys(Boc)-OSu reacted with the amino beads in DCM containing 0.3% TEA overnight. On the following day, the beads were washed with DCM three times, and the Boc protection group was removed by incubation of beads in 50% TFA in DCM at room temperature for 2 h. The beads were washed with DCM three times and one time with 3% TEA in DCM. To continue the derivatization, Boc-Lys(Boc)-OSu was added to the bead DCM solution followed by the addition of TEA (final concentration 3.0%). The reaction was carried out at room temperature with end-over-end rotation overnight. Then the Boc group was deprotected by 50% TFA as mentioned above. The Boc-Lys(Boc)-OSu conjugation step was repeated twice. Then the Fmoc groups were removed by mixing the functionalized beads in 50% piperidine DCM solution at room temperature for 30 min. Finally, all free amine groups were coupled with 5-carboxybenzoboroxole through EDC HOAt chemistry as described above.

### Yeast cell culture and protein extraction

Yeast cells (strain BY4742, MAT alpha, derived from S288c) were grown in yeast extract peptone dextrose (YPD) media until they reached log-phase (optical density (OD) was about 1.0 at 600 nm). For biological duplicate experiments, cells were grown independently. Yeast cells were harvested by centrifugation and resuspended in a buffer containing 50 mM 4-(2-hydroxyethyl)-1-piperazineethanesulfonic acid (HEPES), pH 7.4, 150 mM NaCl, 0.5% sodium deoxycholate (SDC), and protease inhibitor cocktail (one tablet (complete mini, Roche) per 10 ml of lysis buffer) at 4 °C. Cells were lysed using the MiniBeadbeater (Biospec) at maximum speed, three cycles of 30 s each, with 2-min pauses between cycles to avoid overheating the lysates. After centrifugation, lysates were transferred to new tubes, and the protein concentration in the lysate was determined by BCA protein assay (Pierce).

### Human cell culture and protein extraction

MCF7, HEK 293T, and Jurkat cells (American Type Culture Collection (ATCC)) were cultured following the instructions provided by ATCC. Once MCF7 and HEK 293T cells reached 80% confluency, cells were washed with PBS twice and harvested by scraping. Jurkat cells were harvested by centrifugation and then washed with PBS. Cell pellets were suspended in ice-cold RIPA buffer (50 mM HEPES, pH = 7.4, 150 mM NaCl, 0.5% SDC, benzonase (25 U/mL), and protease inhibitor cocktail) and incubated end-over-end for 1 h at 4 °C. After complete solubilization of nuclei and digestion of genomic DNA, the lysate was centrifuged at 25,000 × *g* for 10 min. The supernatant was collected and the protein concentration was measured by BCA protein assay.

### Protein extraction from mouse brain tissues

For mouse brain samples, brain tissues from two C57BL/6 mice (3 and 6 months) were frozen in liquid nitrogen and homogenized in the RIPA buffer mentioned above. The mixtures were incubated on ice for an hour, and then clarified by centrifugation at 5000 × *g* for 20 min. Half of the supernatants (~8 mg of proteins per experiment) were used for protein glycosylation analysis. The resulting proteins from the mouse brain tissues were kindly provided by the laboratory of Dr. Hang Shi at Georgia State University, and all animal experiments were approved by the Georgia State University Institutional Animal Care and Use Committee.

### Protein alkylation and digestion

Lysates from yeast, human cells, or mouse brain tissue were reduced with 5 mM dithiothreitol (DTT) (56 °C, 25 min) and alkylated with 15 mM iodoacetamide (RT, 30 min in the dark). Proteins were purified by the methanol–chloroform precipitation method. The purified proteins were digested with Lys-C (Wako) at a protein:enzyme ratio of ~100:1 in 50 mM HEPES, pH = 8.2, 1.6 M urea, 5% ACN at 31 °C overnight, and then 10 ng/μL trypsin (Promega) for 4 h. Digestion was quenched by the addition of TFA to a final concentration of 0.1%, and the precipitate was removed by centrifugation at 5000 × *g* for 10 min. The supernatant was collected, and peptides were purified using a Sep-Pak tC18 cartridge (Waters).

### Glycopeptide enrichment

For boronic acid derivative experiments, mammalian peptides were dissolved in 100 mM ammonium acetate buffer and incubated for 1 h with different boronic acid-derivatized magnetic beads at room temperature. After incubation, the beads were washed with the binding buffer, and enriched glycopeptides were eluted first by incubation with a solution containing ACN:H_2_O:TFA (50:49:1) at 37 °C for 30 min. Then the glycopeptides were eluted two more times through incubation with 5% formic acid at 56 °C for 5 min each time. For the enrichment of glycopeptides from yeast, human cells, or mouse brain tissues using DBA, ~10 mg of peptides were used in each experiment and incubated with DBA beads in DMSO containing 0.5% TEA, and then washed five times using a buffer containing 50% DMSO and 50% 100 mM ammonium acetate (pH = 11). Glycopeptides were then eluted as described above.

For lectin enrichment, ConA and WGA-conjugated agarose beads (Vector Laboratories) were washed five times using the enrichment buffer (20 mM Tris-base, pH = 7.4, 0.15 M NaCl, 1 mM MgCl_2_, 1 mM CaCl_2_, and 1 mM MnCl_2_)^35^. Peptides were dissolved in the enrichment buffer, mixed with the lectin beads, and vortexed at 37 ^o^C for an hour. The beads were then washed again with the enrichment buffer five times before glycopeptide elution using the elution buffer (0.2 M α-methyl mannoside, 0.2 M α-methyl glucoside, 0.2 M galactose, and 0.5 M *N*-acetyl-d-glucosamine in PBS). The elution was performed twice with vortexing for half an hour each, and the eluents were combined.

For HILIC enrichment, SeQuant® ZIC-HILIC SPE cartridges (the Nest Group) were washed with ten column volumes of 1.0% TFA in water, followed by three washes with the loading buffer (1.0% TFA in 80% ACN, 20% H_2_O)^38–40^. Peptides were loaded onto the column in the loading buffer using a slow flow rate. The column was then washed with the loading buffer three times. Glycopeptides were eluted using 1.0% TFA in water three times, and the eluents were combined.

### Glycopeptide PNGase F treatment and fractionation

The enriched samples were dried in a lyophilizer overnight. The completely dried samples were dissolved in 40 mM ammonium bicarbonate in heavy-oxygen water (H_2_^18^O) and treated with PNGase F (lyophilized powder from Sigma Aldrich) at 37 °C for 3 h. For optimization experiments, after deglycosylation, peptide samples were purified using a stage tip. For all other experiments, enriched glycopeptides were desalted using a tC18 Sep-Pak cartridge, and then subjected to fractionation using high-pH reversed-phase HPLC (pH = 10). The sample was separated into ten fractions using a 4.6 × 250-mm 5-μm particle reversed-phase column (Waters) with a 40-min gradient of 5–50% ACN with 10 mM ammonium acetate. Every fraction was further purified with stage tip before LC–MS/MS.

### LC–MS/MS analysis

Fractionated and purified peptide samples were resuspended in a solvent of 5.0% ACN and 4.0% FA, and 4 μL was loaded onto a microcapillary column packed with C18 beads (Magic C18AQ, 3 μm, 200 Å, 75 μm × 16 cm) using a WPS-3000TPLRS autosampler (UltiMate 3000 Thermostatted Rapid Separation Pulled Loop Wellplate Sampler, Dionex). Peptides were separated by reversed-phase chromatography using an UltiMate 3000 binary pump with a 90-min gradient of 4–30% ACN (in 0.125% FA) and detected in a hybrid dual-cell quadrupole linear ion trap–orbitrap mass spectrometer (LTQ Orbitrap Elite, ThermoFisher) using a data-dependent Top20 method. For each cycle, one full MS scan (resolution: 60,000) in the Orbitrap at 10^6^ AGC target was followed by up to 20 MS/MS in the LTQ for the most intense ions. The isolation window was 2 Da, which is the most commonly used, and the activation energy was 40% normalized collision energy (NCE), which was obtained through testing different NCEs to acquire the best results for the machine used here. Selected ions were excluded from further analysis for 90 s. Ions with a single or unassigned charge were not sequenced. Maximum ion accumulation times (maximum IT) were 1000 ms for each full MS scan and 50 ms for MS/MS scans. For protein *O*-glycosylation analyses, the data were collected using a Q-Exactive Plus Orbitrap mass spectrometer with a 2-h LC gradient. HCD was used as the fragmentation method with the following parameters: 10^6^ AGC target for full MS and 2*10^5^ AGC target for MS^2^, 100 ms maximum IT, 2.0 Da isolation window, and 30% NCE. The dynamic exclusion time was set to 60 s. Both full MS and MS^2^ were collected in the Orbitrap cell with high mass accuracy and high resolution, which contribute to confident identification of *O*-glycopeptides.

### Database searching and data filtering

The raw files were converted into mzXML format prior to the database searching. The SEQUEST algorithm^67^ (version 28) was used to search all MS/MS spectra against either a database containing sequences of yeast (*Saccharomyces cerevisiae*) proteins downloaded from SGD (http://www.yeastgenome.org/), mouse (*Mus musculus*) or human (*Homo sapiens*) proteins downloaded from UniProt. The following parameters were used for the database search: 10-ppm precursor mass tolerance; 1.0-Da product ion mass tolerance; fully tryptic digestion; and up to two missed cleavages; variable modifications: oxidation of methionine (+15.9949) and ^18^O tag of Asn (+2.9883); and fixed modifications: carbamidomethylation of cysteine (+57.0214). In order to estimate the FDR of peptide identification, both forward and reverse orientations of each protein sequence were listed in the database, and the target–decoy method was employed^58^. To distinguish between correct and incorrect peptide identifications, linear discriminant analysis (LDA) was utilized with several parameters such as XCorr, ΔCn, and precursor mass error^68^. After scoring, peptides shorter than seven amino acid residues were discarded, and the remaining peptide spectral matches were controlled to have less than 1.0% FDR. When determining FDRs of the final data set, only glycopeptides were considered.

For *O*-glycopeptide identification, we used Byonic^TM^ software. Some parameters are similar as above. For yeast intact *O*-glycopeptide analysis, up to ten mannoses per glycan were searched for raw files. In order to control false-positive rates, every peptide was required to have ≤0.001 for 1D PEP (one-dimensional posterior error probability) and >4 for |Log Prob| (the absolute value of the log10 of the posterior error probability)^69^. The score of identified glycopeptides must be higher than 300, and the mass accuracy is less than 10 ppm. The PEP takes into account ten features, including the Byonic^TM^ score, delta score, precursor mass error, digestion specificity, etc. Requiring |Log Prob| to be larger than 4 means the *P* value is <10^−4^, based on Neyman–Pearson hypothesis testing. These are very stringent criteria for filtering. For example, for protein *O*-GlcNAcylation analysis, after filtering, there was no reverse hit in the final data sets. For glycoproteins identified in each type of cells, we performed subcellular compartment analysis based on the protein location information downloaded from Uniprot (uniprot.org).

### Protein glycosylation site localization

In order to evaluate the confidence of the glycosylation site assignment, a Modscore was calculated for each of the identified glycopeptides, which is similar to Ascore^70^. An algorithm considering all possible glycosylation sites of a peptide was used to generate the Modscore. It examines the presence or absence of MS/MS fragment ions unique to each glycosylation site and indicates the likelihood that the best site match is correct when compared with the next best match. Sites with Modscore ≥ 19 (*P* ≤ 0.01, cumulative binomial probability) were considered to be confidently localized.

### Data availability

The data sets generated during the current study are available in the PeptideAtlas repository (Dataset Identifier: PASS00980; Password: KV788a), https://db.systemsbiology.net/sbeams/cgi/PeptideAtlas/PASS_View?identifier=PASS00980. In total, there are 142 raw files (20 files for the yeast *N*-glycosylation duplicate experiments, 20 files for the MCF7 *N*-glycosylation duplicate experiments, 10 files for the HEK 293T *N*-glycosylation experiments, 10 files for the Jurkat *N*-glycosylation experiments, 22 files for the mouse brain *N*-glycosylation duplicate experiments, 20 files for the yeast *O*-mannosylation duplicate experiments, 20 files for the MCF7 *O*-GlcNAcylation duplicate experiments, 10 files for the HEK 293T *O*-GlcNAcylation experiments, and 10 files for the Jurkat *O*-GlcNAcylation experiments).

## Electronic supplementary material

Supplementary Information

Description of Additional Supplementary Files

Supplementary Data 1

Supplementary Data 2

Supplementary Data 3

Supplementary Data 4

Supplementary Data 5

Supplementary Data 6

Supplementary Data 7

Supplementary Data 8

Supplementary Data 9

Supplementary Data 10

Supplementary Data 11
