# Supplementary material for: An enrichment method based on synergistic and reversible covalent interactions for large-scale analysis of glycoproteins
Source: Nat Commun. 2018 Apr 27;9:1692. doi: 10.1038/s41467-018-04081-3 (PMC5923262; doi:10.1038/s41467-018-04081-3)
Supplement: Supplementary file 1 — Supplementary Information [file 41467_2018_4081_MOESM1_ESM.docx]

**An enrichment method based on synergistic and reversible covalent interactions for large-scale analysis of glycoproteins**

**Xiao et al.**

**Supplementary Information**

**Supplementary Figures**

**Supplementary Figure 1: Synthesis of the dendrimer with functional amine groups.** Lysine molecules are used as the building blocks to construct the dendrimer on the amine-derivatized magnetic bead.

**Supplementary Figure 2: Conjugation of benzoboroxole to the dendrimer.** After synthesizing the dendrimer on the magnetic beads, benzoboroxole molecules are conjugated to the dendrimer.

**Supplementary Figure 3: The specificity of glycopeptide enrichment in correlation to the DBA bead size.** The specificity of the N-glycopeptide identifications increases with the number of the dendrimer synthesis cycles, and it levels off after the fourth cycle. The overall specificity of glycopeptide enrichment should be higher considering that O-glycopeptides were also enriched.

**Supplementary Figure 4: Effect of solvents on glycopeptide enrichment from a human cell lysate (HEK 293T)**. Light blue - total glycopeptides, dark blue - unique glycopeptides and green - glycoproteins.

**Supplementary Figure 5: Washing buffer optimization for glycopeptide enrichment**. Light blue - total glycopeptides, dark blue - unique glycopeptides and green - glycoproteins.

**Supplementary Figure 6: The effect of washing times on glycopeptide enrichment.** Light blue - total glycopeptides, dark blue - unique glycopeptides and green - glycoproteins.

**Supplementary Figure 7:** **Evaluation of the effect of sample size on the identification of glycopeptides and glycoproteins with the DBA enrichment followed by LC-MS analysis.** Blue - unique glycopeptides, and green - glycoproteins. The error bar represents the standard error of the mean calculated from duplicate experiments.

**Supplementary Figure 8: Duplicate experimental results for assessing residual N-glycans after PNGase F treatment.** Only about 2% N-glycopeptides contained residual glycans after the three-hour treatment (green - unique glycan-containing peptides, and blue - unique deglycosylated peptides).

**Supplementary Figure 9: Comparison of N-glycoproteins identified in yeast cells in duplicate experiments.**

**Supplementary Figure 10: Comparison of the abundance distributions of yeast N-glycoproteins identified in this work (blue) and identified previously with the phenylboronic acid beads in 2014 (green)^1^**.

**Supplementary Figure 11: MS parameters were optimized for yeast intact O-glycopeptide analysis using the Orbitrap cell to record tandem mass spectra of glycopeptides.** (**a**) AGC target for full MS, (**b**) AGC target for MS^2^ (tandem mass spectrum), (**c**) comparison of Top10 (selection of the 10 most abundant ion species) and Top15 (selection of the 15 most abundant ion species) methods, (**d**) normalized collision energy, (**e**) maximum ion accumulation time for MS^2^. The error bar represents the standard error of the mean calculated from duplicate experiments.

**Supplementary Figure 12: Examples of O-mannosylated peptides identified in this work.** (**a**) Glycopeptide ANSLNELDVTATT[Hex_9_]VAK from protein GAS3. (**b**) Glycopeptide SYSAT[Hex_8_]TSDVACPATGK from protein GAS1. (**c**) Glycopeptide FSSSLS [Hex_5_]AQAFPR from protein EXG2. (**d**) Glycopeptide ISASSIDAS[Hex_7_]GFVQK from protein SED4. (**e**) Glycopeptide TLDDFNNYS[Hex_6_]SEINK from protein GAS1. (**f**) Glycopeptide YPEAGPTAPVT[Hex_2_]K from protein YD056. (**g**) Glycopeptide DDTIS [Hex_4_]ATISYDK from protein GAS3. (**h**) Glycopeptide VENGQTLT[Hex_6_]TFITK from protein PRY2. Hex stands for hexose residue(s).

**Supplementary Figure 13: Clustering of identified O-glycoproteins in yeast based on** **molecular function.** *P* values were calculated using a modified Fisher’s exact test.

**Supplementary Figure 14: Comparison of N-glycoproteins identified in MCF7 cells from duplicate experiments.**

**Supplementary Figure 15: Comparison of N-glycosylation sites identified in MCF7, HEK 293T and Jurkat cells.**

**Supplementary Figure 16: The distribution of unique N-glycosylation sites per glycoprotein in human cells.**

**a**

**b**

**Supplementary Figure 17: Clustering of N-glycoproteins based on (a) molecular function and (b) cellular compartment.** *P* values were calculated using a modified Fisher’s exact test.

**Supplementary Figure 18: The number of receptors (N-glycoproteins) identified in each type of human cells.**

**Supplementary Figure 19: The number of protein N-glycosylation sites (a) and glycoproteins (b) identified in mouse brain tissues from biological duplicate experiments.**

**Supplementary Figure 20: Clustering of glycoproteins identified in mouse brain tissues based on biological process.** *P* values were calculated using a modified Fisher’s exact test.

**Supplementary Figure 21: CD N-glycoproteins identified in three types of human cells.** The numbers of CD N-glycoproteins (**a**), and the percentage of CD glycoproteins with respect to all N-glycoproteins (**b**) identified in each type of human cells.

**Supplementary Figure 22: Two examples of glycoproteins (CD30 and CD96) with domain and glycosylation site information in Jurkat cells.**

.

**SUPPLEMENTARY NOTES**

**Supplementary Note 1: Boronic acid derivatives to enhance enrichment interactions**

Reversible interactions between boronic acid and sugars have great potential to enrich glycopeptides/glycoproteins^1-3^. For global analysis of protein glycosylation, enrichment through strong interactions between boronic acid and glycopeptides is critical to cover low-abundance glycopeptides.

There are several major factors that govern the interactions between boronic acid (BA) and sugars, including the p*K_a_* of BA, the solution pH, and steric/stereoelectronic effects^4, 5^. Although BA with a lower p*K_a_* is expected to have greater binding affinities at neutral pH, this is not always true for glycopeptide enrichment. Therefore, several BA derivatives with various p*K_a_* values were tested, and the optimal pH was found for each BA derivative. Previously, we demonstrated that phenylboronic acid conjugated beads were able to enrich glycopeptides from yeast whole cell lysates^1^. In yeast, high mannose glycans dominate, while glycans are more structurally diverse in mammalian cells. Here, we have designed and optimized a BA-based method to effectively enrich glycopeptides from mammalian cell lysates. The structures of several BA derivatives tested here are displayed in Fig. 1a. Each of these BA derivatives was conjugated to magnetic beads containing either carboxylate or amine groups. After the -NH_2_ or -COOH group reacts with the corresponding groups on the magnetic beads, the amide bond (-CONH-) between the beads and the benzene ring in each BA derivative should have a minimal effect on the optimal binding pH values.

In parallel experiments starting with the same amount of purified peptides from human cells (HEK 293T), we examined these BA derivatives at different pH values and compared the number of unique identified N-glycopeptides. Very few glycopeptides were identified at pH=7 or 8 with any BA derivative. For all derivatives, the optimal pH was 10 or 11, as shown in Fig. 1b. The derivatives **IV** and **V** enriched slightly more unique glycopeptides compared to phenylboronic acid (**III**). Although the p*K_a_* of derivative **I** (9.2) is similar to that of phenylboronic acid (9.0), enrichment with **I** resulted in the identification of more unique glycopeptides. One possible reason is that the adjacent nitrogen may form an extra hydrogen bond with a nearby hydroxyl group on the glycan, which enhances the interactions between the BA derivative and glycans and facilitates the enrichment.

**Supplementary Note 2: Further optimization of experimental conditions for DBA enrichment**

*Effect of different solvents on glycopeptide enrichment*. A variety of solvent combinations were tested for glycopeptide enrichment with DBA beads (Supplementary Fig. 4). The pH of all aqueous solutions was adjusted to 11 using an ammonium acetate buffer. For each combination, the binding step of enrichment was performed for an hour in the first solution, and then the beads were washed five times in the second solution. The combination of “DMSO+DMSO” provided the highest enrichment efficiency with the identification of the most N-glycopeptides and glycoproteins. This is consistent with *Le Chatelier*'s principle because water is the product of the reaction between the boronic acid derivative and sugars. Without water, the reaction shifts toward the direction of bond formation and becomes more complete.

*Washing buffer for glycopeptide enrichment optimization*. Based on the results from Supplementary Fig. 4, several washing buffers were tested, and the results are in Supplementary Fig. 5. We performed the enrichment in DMSO containing 0.5% trimethylamine (TEA) for one hour, and then washed the beads with different buffer combinations. The enriched peptides were subsequently deglycosylated and analyzed by LC-MS/MS. The washing buffer containing 50% DMSO and 50% H_2_O (pH=11) outperformed all other combinations. The addition of water helped remove non-specifically bound peptides and increased the number of identified glycopeptides and glycoproteins.

*Number of washes for glycopeptide enrichment optimization*. Based on the previous results, we further optimized the number of washes (0-9 washes). All parallel experiments started with about 0.25 mg mammalian peptides, which were enriched with the DBA beads in DMSO containing 0.5% TEA for one hour, and then the number of times the beads were washed with 50% DMSO and 50% H_2_O (pH=11) was varied. From 0 to 4 washes, a linear trend was found for N-glycopeptide and glycoprotein identifications because increasing the number of washes removed non-specifically bound peptides. After washing four times, there was no obvious change (Supplementary Fig. 6). These results indicate that the interactions between DBA and glycans are very strong because washing more times did not result in the loss of glycopeptides.

*Effect of the sample size on the identification of glycopeptides and glycoproteins*. Different amount of cultured MCF 7 cells were used to evaluate the sample size effect on the N-glycopeptide identification with the DBA enrichment. Duplicate experiments were performed. Cells in each group were harvested and the final protein amounts in the eight groups were around 10, 30, 60, 100, 200, 300, 500, and 1000 µg, respectively. After protein precipitation and digestion, the peptides were subject to DBA enrichment. The enriched glycopeptides were then purified and analyzed by LC-MS/MS. The data is presented in Supplementary Fig. 7.

The lowest number of glycoproteins we identified in one MS run was about 200 from the 10 µg group among the samples tested here. When the sample amount is very small, the sample loss coming from every step may be a problem. For instance, when a very small volume of solvent (lysis buffer or digestion buffer) was used for cell lysis and protein digestion, the sample transfer from tube to tube could result in a considerable (relatively higher percentage) sample loss. More samples allowed us to identify higher numbers of unique glycopeptides and glycoproteins. After the protein amount reached ~300 µg, the increasing trend of the number of identified glycopeptides and glycoproteins slowed down, and both the 500 µg and 1000 µg groups yielded almost the same results. Besides the sample loss, the MS speed and sensitivity may also dramatically affect the number of unique glycopeptides identified. A machine with higher speed and sensitivity allows us to identify more glycoproteins using the same amount of material or the same number of glycoproteins using a lower amount of material. Of note, normally the protein digestion efficiency and peptide purification efficiency are lower than 100%, and therefore, the resulting peptide amounts subjected to the DBA enrichment in the current experiment should be slightly lower than the sample amounts shown in the figure.

**Supplementary Note 3: Evaluation of the PNGase F treatment efficiency***.*

In regard to assessing the extent of residual N-glycans after PNGase F treatment, we performed duplicate experiments to examine the percentage of residual N-glycans (Supplementary Fig. 8). The results indicated that the removal of N-glycans with PNGase F was effective. Briefly, peptides from MCF7 whole cell protein digestion were subject to enrichment with the DBA beads. The enriched glycopeptides were then treated with PNGase F in H_2_^18^O for three hours. The purified peptides were analyzed using an online LC-MS/MS system with a Q-Exactive Plus mass spectrometer, and both full MS and MS/MS were recorded in the Orbitrap cell. Higher-energy collisional dissociation (HCD) was used as the fragmentation method. We searched for the deglycosylated peptides (2.9883 Da mass shift on N) and the N-glycan-containing peptides using Byonic. As a result, 44 unique glycan-containing peptides and 1,866 deglycosylated peptides were identified in the first experiment; 45 unique glycan-containing peptides and 1,871 unique deglycosylated peptides were identified in the second experiment. Overall, N-glycopeptides with residual N-glycans are only around ~2%, demonstrating that the three-hour PNGase F treatment was effective to remove N-glycans.

We performed the control experiments previously^6^ and found that the effect of spontaneous deamidation was negligible under the treatment conditions (pH=7.5 and 37 ^o^C) for three hours. For all our experiments for protein N-glycosylation analysis, we strictly controlled the treatment time within three hours. Although a longer treatment time may lead to more complete removal of N-glycans and result in the identification of more N-glycosylation sites, spontaneous deamidation will cause higher false positive rates for protein N-glycosylation site identification.

**Supplementary Note 4: Two examples of CD glycoproteins identified in Jurkat cells**

Two examples of glycoproteins identified in Jurkat cells are shown in Supplementary Fig. 22. TNFRSF8, also called CD30, is a receptor for TNFSF8/CD30L and may play a role in the regulation of cellular growth and transformation of activated lymphoblasts. Here, we identified three N-glycosylation sites (N101, N120 and N295), and all these sites were located in the TFNR domain, which is well-known to bind growth factors, and glycans on these domains may affect the binding. CD96 is T-cell surface protein tactile and may be involved in adhesive interactions of activated T and NK cells during the late phase of the immune response^7^. It contains three Ig domains (Ig-like V type 1, Ig-like V type 2 and Ig-like C2-type). Six out of the eight N-glycosylation sites were identified in two domains, and all sites are located in the extracellular space. Glycosylation sites identified on CDs may carry more meaningful information for cell classification and serve as effective biomarkers for disease detection.

**SUPPLEMENTARY REFERENCES**

1. Chen, W.X., Smeekens, J.M. & Wu, R.H. A Universal chemical enrichment method for mapping the yeast N-glycoproteome by mass spectrometry (MS). *Molecular & Cellular Proteomics* **13**, 1563-1572 (2014).

2. Sparbier, K., Wenzel, T. & Kostrzewa, M. Exploring the binding profiles of ConA, boronic acid and WGA by MALDI-TOF/TOF MS and magnetic particles. *J. Chromatogr. B* **840**, 29-36 (2006).

3. Xu, G.B., Zhang, W., Wei, L.M., Lu, H.J. & Yang, P.Y. Boronic acid-functionalized detonation nanodiamond for specific enrichment of glycopeptides in glycoproteome analysis. *Analyst* **138**, 1876-1885 (2013).

4. Jin, S., Cheng, Y.F., Reid, S., Li, M.Y. & Wang, B.H. Carbohydrate recognition by boronolectins, small molecules, and lectins. *Med. Res. Rev.* **30**, 171-257 (2010).

5. Peters, J.A. Interactions between boric acid derivatives and saccharides in aqueous media: Structures and stabilities of resulting esters. *Coord. Chem. Rev.* **268**, 1-22 (2014).

6. Xiao, H.P., Tang, G.X. & Wu, R.H. Site-specific quantification of surface N-glycoproteins in statin-treated liver cells. *Analytical Chemistry* **88**, 3324-3332 (2016).

7. Fuchs, A., Cella, M., Giurisato, E., Shaw, A.S. & Colonna, M. Cutting edge: CD96 (Tactile) promotes NK cell-target cell adhesion by interacting with the poliovirus receptor (CD155). *J. Immunol.* **172**, 3994-3998 (2004).
