# Supplementary material for: An enrichment method based on synergistic and reversible covalent interactions for large-scale analysis of glycoproteins
Source: Nat Commun. 2018 Apr 27;9:1692. doi: 10.1038/s41467-018-04081-3 (PMC5923262; doi:10.1038/s41467-018-04081-3)
Supplement: Supplementary file 2 — Description of Additional Supplementary Files [file 41467_2018_4081_MOESM2_ESM.pdf]

Description of Additional Supplementary Files

File Name: Supplementary Data 1
Description: Protein N-glycosylation sites identified in duplicate yeast Experiments

File Name: Supplementary Data 2
Description: Unique O-glycopeptides identified in duplicate yeast Experiments

File Name: Supplementary Data 3
Description: Protein N-glycosylation sites identified in duplicate experiments in MCF7 cells

File Name: Supplementary Data 4
Description: Protein N-glycosylation sites identified in HEK 293T cells

File Name: Supplementary Data 5
Description: Protein N-glycosylation sites identified in Jurkat cells

File Name: Supplementary Data 6
Description: Protein N-glycosylation sites (4691) identified in MCF7, HEK 293T and Jurkat cells

File Name: Supplementary Data 7
Description: A total of 1906 N-glycoproteins identified in MCF7, HEK 293T and Jurkat cells

File Name: Supplementary Data 8
Description: Receptors (301) containing N-glycosylation sites identified in MCF7, HEK 293T and Jurkat cells

File Name: Supplementary Data 9
Description: Protein N-glycosylation sites identified in the mouse brain Tissues

File Name: Supplementary Data 10
Description: O-glycoproteins with one HexNAc identified in three types of human cells

File Name: Supplementary Data 11
Description: CDs identified in three types of human cells
